# MicroRNAs miR-4535 and miR-1915-5p in amniotic fluid as predictive biomarkers for chorioamnionitis

**DOI:** 10.2144/fsoa-2021-0006

**Published:** 2021-02-15

**Authors:** Chihiro Kiyoshima, Naoto Shirasu, Daichi Urushiyama, Satoshi Fukagawa, Toyofumi Hirakawa, Kenichi Yoshikawa, Daisuke Izuchi, Kohei Miyata, Masamitsu Kurakazu, Fusanori Yotsumoto, Kenji Hiromatsu, Makoto Nomiyama, Ohta Eiji, Shinichi Hirose, Yoshitoshi Ogura, Tetsuya Hayashi, Kenichiro Hata, Kazuki Nabeshima, Shin'ichiro Yasunaga, Shingo Miyamoto

**Affiliations:** 1 Department of Obstetrics & Gynecology, Fukuoka University, Fukuoka, Japan; 2 Department of Biochemistry, Fukuoka University, Fukuoka, Japan; 3 Central Research Institute for Advanced Molecular Medicine, Fukuoka University, Fukuoka, Japan; 4 Department of Microbiology & Immunology, Faculty of Medicine, Fukuoka University, Fukuoka, Japan; 5 Department of Obstetrics & Gynecology, National Hospital Organization Saga National Hospital, Saga, Japan; 6 Center for Maternal, Foetal & Neonatal Medicine, Fukuoka University Hospital, Fukuoka, Japan; 7 Departemnt of Pediatrics, Faculty of Medicine, Fukuoka University, Fukuoka, Japan; 8 Department of Bacteriology, Faculty of Medical Sciences, Kyushu University, Fukuoka, Japan; 9 Department of Maternal-Fetal Biology, National Research Institute for Child Health & Development, Tokyo, Japan; 10 Department of Pathology, Fukuoka University School of Medicine & Hospital, Fukuoka, Japan

**Keywords:** 16S ribosomal DNA, amniotic fluid, chorioamnionitis, ddPCR, miR-1915-5p, miR-4353, next-generation sequencing

## Abstract

**Background:**

This study was performed to investigate the clinical significance of miR-4535 and miR-1915-5p in severe chorioamnionitis.

**Materials & methods:**

Amniotic fluid samples from 37 patients with severe chorioamnionitis were subjected to miRNA array analysis and ddPCR™. Diagnostic values were assessed using the receiver operating characteristic curve. The patients were separated into three groups according to Blanc’s criteria.

**Results:**

The expression of miR-4535 and miR-1915-5p was significantly correlated with the copy number of 16S rDNA, had extremely high diagnostic accuracy for severe chorioamnionitis, and was linked to maternal and fetal inflammation.

**Conclusion:**

miR-4535 and miR-1915-5p serve as promising biomarkers for the diagnosis of severe chorioamnionitis.

## Lay abstract

Chorioamnionitis and fetal inflammatory response syndrome, which is linked to chorioamnionitis, are considered serious diseases in perinatal care. In this study, miR-4535 and miR-1915-5p are recognized as promising biomarkers for the diagnosis of chorioamnionitis before delivery. In particular, the increased expression of miR-4535 in amniotic fluid is expected to be regarded as a positive indicator for fetal inflammatory response syndrome, and the elevated expression of miR-4535 in serum is also considered to predictively diagnose intrauterine infection in pregnancy. Our results highlight that further studies should explore the underlying clinical significance of miR-4535 and miR-1915-5p.

Chorioamnionitis is a perinatal condition characterized by inflammation of the fetal membrane [1] which leads to premature birth, neonatal sepsis and brain diseases in infants [2–5]. It is histologically diagnosed by the presence of neutrophil infiltration in the placental tissues after delivery, the so-called Blanc’s classification [1]. Owing to limitations before delivery, the diagnosis of chorioamnionitis is often done clinically based on the following indicators: maternal fever, uterine pain, maternal tachycardia, fetal tachycardia, maternal leukocytosis and foul-smelling uterine discharge [6]. However, the variability and lack of sensitivity of the clinical diagnosis of chorioamnionitis makes it difficult to predict maternal and fetal outcomes [7,8]. An accurate and easy-to-use diagnostic test to predict chorioamnionitis is therefore required.

Detection of bacterial pathogens or various inflammatory reactions has commonly been used to predict fetal outcomes in patients with severe chorioamnionitis. The detection of bacterial or fungal organisms from amniotic fluid or placental samples has been performed via culturing or PCR-based methods. However, only a small percentage of placental cultures have revealed causative organisms [9]. Although PCR has been successfully used to increase method sensitivity, it still fails to detect certain pathogens [10–12]. Recently, we have demonstrated that the absolute numerical quantification, as well as the metagenomic sequencing, of 16S rDNA in amniotic fluids can be used for the accurate diagnosis of chorioamnionitis [13]. Consequently, we found 11 bacterial species causing intrauterine infection in pregnancy as microbiomic chorioamnionitis (miCAM), and the presence of these bacterial species in amniotic fluid has been significantly associated with severe chorioamnionitis, such as Blanc’s classification stages II–III (stage II: chorionitis; stage III: chorioamnionitis), and adverse perinatal outcomes [13]. These 11 bacterial species consist of indigenous bacteria in the genital tract as well as the oral and digestive tracts. The indigenous bacteria in the genital tract comprise seven species: *Ureaplasma parvum, Streptococcus agalactiae, Gardnerella vaginalis*, *Sneathia sanguinegens, Prevotella bivia, Lactobacillus jensenii* and *Mycoplasma hominis* [13]. There are four indigenous bacteria in the oral and digestive tracts: *Streptococcus anginosus, Eikenella corrodens, Bacteroides fragilis* and *Porphyromonas endodontalis* [13]. The presence of *Ureaplasma* spp. in amniotic fluid induces the risk of developing chorioamnionitis and preterm birth [14,15]. In preterm birth infants, *Ureaplasma spp*. are the most commonly detected microorganisms [16,17]. In addition, among the infection of miCAM (*U. parvum, S. agalactiae, G. vaginalis*, *S. sanguinegens, P. bivia, L. jensenii* and *M. hominis*), we have previously reported that 33 of 43 (77%) patients with severe chorioamnionitis were identified as having *U. parvum* in amniotic fluid, using a next-generation sequencing method [13]. In contrast, inflammatory changes have a greater association with adverse perinatal outcomes than the presence of the aforementioned microorganisms [18–21], thus promising predictive biomarkers for chorioamnionitis must be identified in host inflammatory reactions and via absolute quantification and sequencing of 16S rDNA.

miRNAs are short noncoding RNAs with 21–25 nucleotides that modulate the expression of the target gene in various biological processes, such as cell proliferation, differentiation and death [22–24]. In addition, miRNAs are implicated in various disorders, including cancer, diabetes, cardiovascular and infectious diseases [22–24]. miRNAs exist in nanosized vesicular particles produced in various biofluids and have been recognized as ideal biomarkers for diseases [25–27]. The involvement of miRNAs has been well established in various infectious diseases, including bacterial sepsis, chronic refractory respiratory disease, hepatic diseases and *Plasmodium malaria* infection [28–30]. Accumulating evidence indicates that numerous miRNAs regulate the complex interaction between survival strategies of bacteria and host innate immune pathways [31,32]. In addition to this evidence, miRNAs seem to be recognized as clinically valuable biomarkers at the early stage of diseases or for specific kinds of diseases [33]. Accordingly, we hypothesize that alterations in host miRNAs can be used to indicate the occurrence of chorioamnionitis.

miRNAs play pivotal roles in many stages of pregnancy, including implantation, gestation and labor, and thus may contribute to the maintenance of a healthy pregnancy [34]. The placenta produces abundant miRNAs. In the first trimester, miR-378a-5p enhances the proliferation and expansion of extravillous trophoblast cells at the placental explants [35]. The expression of miR-376c in the placenta from patients with pre-eclampsia is suppressed compared with that in the placentas of normal pregnant women, and the inhibition of miR-376c is linked to the proliferation and invasion of trophoblasts [36]. The miR-17-92 cluster is involved in the differentiation of trophoblasts through the repression of *hGCM1* and *hCYP19A1* [37]. In a case–control study, miR-155 and miR-210 have been implicated as serum biomarkers in pre-eclampsia [38]. In addition to this evidence, some miRNAs in serum have been also reported as potential biomarkers for early diagnosis of placental insufficiency and related issues such as fetal growth restriction and pre-eclampsia [39–41]. Accordingly, many miRNAs have been reported in association with pathophysiological episodes in placentation. Recently, the miRNA profiling of amniotic fluid was reported, suggesting that miRNAs in the amniotic fluid may contribute to fetal development [42]. The relationship between miRNAs in the amniotic fluid and fetal congenital diseases including chromosomal anomalies has also been investigated [43–45]. In chorioamnionitis miR-548, which is highly expressed in the amniotic membrane, alters the inflammatory responses through the regulated expression of *HMGB1*, and miR-223 and miR-338 are also abundantly expressed in the chorioamniotic membranes [46,47]. However, there have been no reports concerning the association between chorioamnionitis and the expression of miRNAs in the amniotic fluid.

In this study we aimed to identify miRNAs in amniotic fluid that may serve as biomarkers for chorioamnionitis. We selected three candidate miRNAs in patients with chorioamnionitis, namely miR-4535, miR-1915-5p and miR-191-5p, and studied the correlation of miRNA expression with the copy number of 16S rDNA.

## Materials & methods

### Histological criteria

Histological chorioamnionitis was diagnosed from findings of acute inflammatory sites in the chorion or amnion in accordance with Blanc’s criteria [1]: for stage III (chorioamnionitis), the neutrophilic infiltrate is found in subamniotic connective tissues as well as the amniotic epithelium; for stage II (chorionitis), the neutrophilic infiltration is detected in the chorionic plate or the membranous chorionic connective tissues; for stage I (subchorionitis), patchy or diffused accumulation of neutrophils is observed in the subchorionic plate or decidua. Patients classified under Blanc’s stages II–III were clinically diagnosed with severe chorioamnionitis.

### miCAM

We defined amniotic fluid samples as miCAM positive when any of the 11 species we had previously identified as markers for prenatal diagnosis of severe chorioamnionitis, which were remarkably predominant in stage III, were most abundant [13]. These 11 bacterial species are indigenous bacteria in the genital tract or the oral and digestive tracts. Seven of these bacterial species, namely *U. parvum, S. agalactiae, G. vaginalis*, *S. sanguinegens, P. bivia, L. jensenii* and *M. hominis*, are indigenous bacteria of the genital tract [13]. The indigenous bacteria of the oral and digestive tracts comprise four bacterial species: *S. anginosus, E. corrodens, B. fragilis* and *P. endodontalis* [13].

### Patients

To first explore the candidate miRNAs in amniotic fluid samples for clinical prognosis, the five groups (α–ε) were split up in the 15 pregnant women for miRNA array analysis (Table 1). Group α consisted of two representative patients with amniotic fluid infected with at least two miCAM species, including *U. parvum*. Group β included three patients with only *U. parvum* infection. Group γ included three patients infected with miCAM species except *U. parvum.* Group δ was composed of four patients infected with non-miCAM species. Group ε comprised three healthy individuals without chorioamnionitis.

**Table 1.** Clinical characteristics of the pregnant participants and the microbiome species present in their amniotic fluid, analyzed using comprehensive miRNA array.

Patient code	Chorioamnionitis^‡^	WBC in maternal peripheral blood (cells/μl)	CRP level in maternal peripheral blood (mg/dl)	Microbiome species (relative abundance [%]) detected						
				miCAM; 11 species^†^						
				*Ureaplasma parvum*	*Streptococcus agalactiae*	*Gardnerella vaginalis*	*Streptococcus anginosus*	*Bacteroides fragilis*	*Mycoplasma hominis*	Others
α1	Stage II	6700	3.0	33.7	–	–	–	–	64.5	–
α2	Stage II	17,900	4.2	44.5	54.5	–	–	–	–	–
β1	Stage III	20,100	2.4	99.9	–	–	–	–	–	–
β2	Stage III	19,600	0.8	100.0	–	–	–	–	–	–
β3	Stage III	13,400	0.7	99.8	–	–	–	–	–	–
γ1	Stage III	19,700	8.6	–	–	99.5	–	–	–	–
γ2	Stage II	14,000	4.8	–	–	99.5	–	–	–	–
γ3	Stage II	7700	0.7	–	–	13.1	9.8	5.9	–	70.9
δ1	Stage II	10,400	3.1	–	–	–	–	–	–	100.0
δ2	Stage II	6400	1.3	–	–	–	–	–	–	100.0
δ3	Stage II	9800	0.1	–	–	–	–	–	–	100.0
δ4	Stage II	7000	0.2	–	–	–	–	–	–	100.0
ε1	Stage 0–1	–	–	–	–	–	–	–	–	100.0
ε2	Stage 0–1	–	–	–	–	–	–	–	–	100.0
ε3	Stage 0–1	–	–	–	–	–	–	–	–	100.0

† *Sneathia sanguinegens*, *Eikenella corrodens*, *Prevotella bivia*, *Lactobacillus jensenii* and *Porphyromonas endodontalis* were not detected.

‡ The staging is based on Blanc’s classification. Stage III (chorioamnionitis): neutrophilic infiltrates in subamniotic connective tissues and the amniotic epithelium; stage II (chorionitis): neutrophilic infiltration of the chorionic plate or membranous chorionic connective tissues; stage I (subchorionitis): patchy or diffused accumulation of neutrophils within the subchorionic plate or decidua.

CRP: C-reactive protein; miCAM: Microbiome chorioamnionitis; WBC: White blood cell.

To subsequently identify the clinical significance of the candidate miRNAs, we analyzed the expression of miRNAs and the copy number of 16S rDNA in the amniotic fluid samples of pregnant women (n = 63). The samples collected for miRNA expression analysis comprised 37 with Blanc’s classification stages II–III (groups II–III), 11 with Blanc’s classification stages 0–I (group I) and 15 at weeks 15–17 of gestation who required amniocentesis for possible fetal chromosome abnormality and agreed to participate in this study (control group). In groups I–III, 21 and 16 patients had miCAM and non-miCAM infection, respectively. To finally investigate the relationship between the expression of miRNAs in the amniotic fluid and serum, we examined the expression of miRNAs and the copy number of 16S rDNA in 14 pregnant women, including 9 with Blanc’s classification stages II–III and miCAM and 5 with Blanc’s classification stages 0–I. The clinical results of the newborn babies were available for 31/37 women in groups II–III.

The Ethics Committee of the Fukuoka University Hospital and the National Hospital Organization Saga National Hospital approved this study (approval numbers 15-2-08 and 23-4). All participants provided written informed consent, and the potential risks were clearly explained to them. Clinical and clinicopathological information for pregnant women was gathered from their clinical records.

### Sample collection

Amniotic fluid and/or serum samples from pregnant women were collected from 2010 to 2018 at the Fukuoka University Hospital (n = 50) and the National Hospital Organization Saga National Hospital (n = 13). In order to obtain the amniotic fluid samples, amniocentesis was performed percutaneously under transabdominal ultrasound guidance in sterile conditions. Maternal serum samples were taken on the same day as amniocentesis. Peripheral blood from infants was obtained during birth. Within 24 h after collection, the amniotic fluid and serum samples were centrifuged at 3000 rpm for 20 min at 25 ± 1°C. The pellet was then separated and removed. The supernatant was stored at -80°C until ready for use for RNA or DNA extraction.

### miRNA microarray

Total RNA was extracted from 300 μl of amniotic fluid using the 3D-Gene^®^ RNA extraction reagent (Toray Industries, Inc., Tokyo, Japan). Half of the extracted RNA was used for comprehensive miRNA expression analysis using a 3D-Gene miRNA labeling kit and a 3D-Gene Human miRNA Oligo Chip (Toray). Scanning was performed on a 3D-Gene Scanner 3000 (Toray). The 3D-Gene extraction software (v. 1.2, Toray) was used to read the raw intensity of the image. The raw data were analyzed using GeneSpringGX (v. 10.0, Agilent Technologies, CA, USA) and quartile normalized. The microarray data from this study have been deposited at the NCBI Gene Expression Omnibus with the accession number GSE143193.

### Total RNA & DNA extraction

Total RNA was isolated from the amniotic fluid and serum samples using a miRNeasy serum/plasma kit (Qiagen, Hilden, Germany). Briefly, 200 μl of the amniotic fluid or serum was mixed with 1.0 ml of Qiazol lysis reagent. The specimen was then allowed to react for 5 min at 25 ± 1°C. For a spike-in control, 3.0 μl of commercially synthesized *Caenorhabditis elegans* miR-39-3p (6.0 × 10^12^ copies/μl, Cel-miR-39-3p; Genenet Co., Ltd, Fukuoka, Japan) was mixed with each specimen. Total RNA was isolated in accordance with the manufacturer’s protocol. The specimen was then completely eluted in 35 μl of RNase-free water. The extracted RNA samples were frozen for preservation at −80°C. Each stored sample of RNA was thawed, and the specimen was lysed in Pathogen Lysis Tubes L (Qiagen). DNA in each sample was allowed to react using a QIAamp UCP Pathogen Mini Kit (Qiagen) in accordance with the manufacturer’s protocol as described previously [13].

### RT-PCR & ddPCR

For cDNA synthesis, 5 μl of total RNA was allowed to react with specific and respective primers for miRNAs and Cel-miR-39-3p using a TaqMan™ MicroRNA reverse transcription kit (Applied Biosystems, CA, USA), in accordance with the manufacturer’s protocol. The products were stored at -40°C.

Quantification of miRNA expression was performed using ddPCR™ using TaqMan^®^ miRNA assays. cDNA (2 μl) was mixed with 18 μl of QX200 reagent (Bio-Rad, CA, USA). Each specimen was then divided into approximately 20,000 droplets using a QX200 Droplet Generator (Bio-Rad). PCR was conducted under the following conditions in accordance with the manufacturer’s protocol: incubation for 5 min at 95°C, 40 cycles of 30 s at 95°C and 1 min at 52°C, and incubation for 5 min at 4°C, followed by incubation for 5 min at 90°C. The PCR temperature was decreased to 4°C at a rate of 2°C/s (annealing temperature: 52°C). Fluorescence analysis was performed using a QX200 Droplet Reader (Bio-Rad) and quantified using the Bio-Rad QuantaSoft software. Copy numbers of the miRNAs in 1 μl of the sample were estimated. The expression of miRNA was normalized to that of the synthetic spike-in, miR-39-3p. For the TaqMan primers and probes (Applied Biosystems), the following were used: hsa-miR-1915-5p, 121120_mat; hsa-miR-4535, 464284_mat; hsa-miR-191-5p, 002299; cel-miR-39, 000200; hsa-miR-208a-5p, 462036_mat; hsa-miR-345-3p, 474987_mat; hsa-miR-6807-5p, 466930_mat; hsa-miR-6769a-5p, 467103_mat; hsa-miR-7854-3p, 466691_mat; and hsa-miR-6501-3p, 476038_mat.

To accurately quantify the 16S rDNA, ddPCR was performed using the EvaGreen dye with universal primers (27Fmod and 338R) (Sigma-Aldrich Japan, Tokyo, Japan) in order to assess bacterial load, as described previously [13]. Extracted DNA (1 μl) was completely diluted and allowed to react with 19 μl of QX200 reagent. Each sample was divided into approximately 20,000 droplets using a QX200 Droplet Generator. PCR was performed under the following conditions in accordance with the manufacturer’s protocol: incubation for 5 min at 95°C, 40 cycles of 30 s at 95°C and 1 min at 60°C, and incubation for 5 min at 4°C, followed by incubation for 5 min at 90°C. The PCR temperature was decreased to 4°C at a rate of 2°C/s (annealing temperature: 60°C). Similar to the TaqMan miRNA assay, fluorescence analysis was performed using a QX200 Droplet Reader. The copy number of each 16S rDNA in 1 ml of the sample was then measured.

### Target gene profiling *in silico*

To understand the functions of the miRNAs, we performed miRNA target prediction using DIANA-microT-CDS algorithms (http://diana.imis.athena-innovation.gr), miRDB (http://mirdb.org/miRDB/) and Targetscan (http://www.targetscan.org/vert_71/) in terms of base sequence. Additionally, we conducted all prediction processes using custom-written executable files in order to compute the parameters of miRNAs for mRNAs on the basis of inherent algorithms and set thresholds. The thresholds for the algorithms indicated an miTG score >0.8 for DIANA-microT-CDS, target score ≥60 for miRDB and context score ≤0.4 for Targetscan.

### Statistical analysis

Mann–Whitney U test or Dunn’s multiple comparison test was applied to determine statistical significance in multigroup comparisons, whereas the Student *t* test was used for pairwise comparison. All experiments were conducted in triplicate, and data are expressed as the mean ± standard error of the mean. The cutoff values of miR-4535, miR-1915-5p and miR-191-5p expression were the median of the expression in amniotic fluids among 63 pregnant women. The correlation is presented as the Pearson correlation coefficient (*r*). In order to evaluate diagnostic significance, we constructed receiver operating characteristic (ROC) curves. In addition, we calculated the AUC and Youden index. Pearson’s χ-square test was used. Statistical significance was established at p < 0.05. Statistical analyses were performed using GraphPad Prism v8.0 (GraphPad Software, CA, USA) and SPSS v16.0J (SPSS Japan, Tokyo, Japan) for Windows.

## Results

### Exploration of candidate miRNAs associated with severe chorioamnionitis

**Figure 1.**
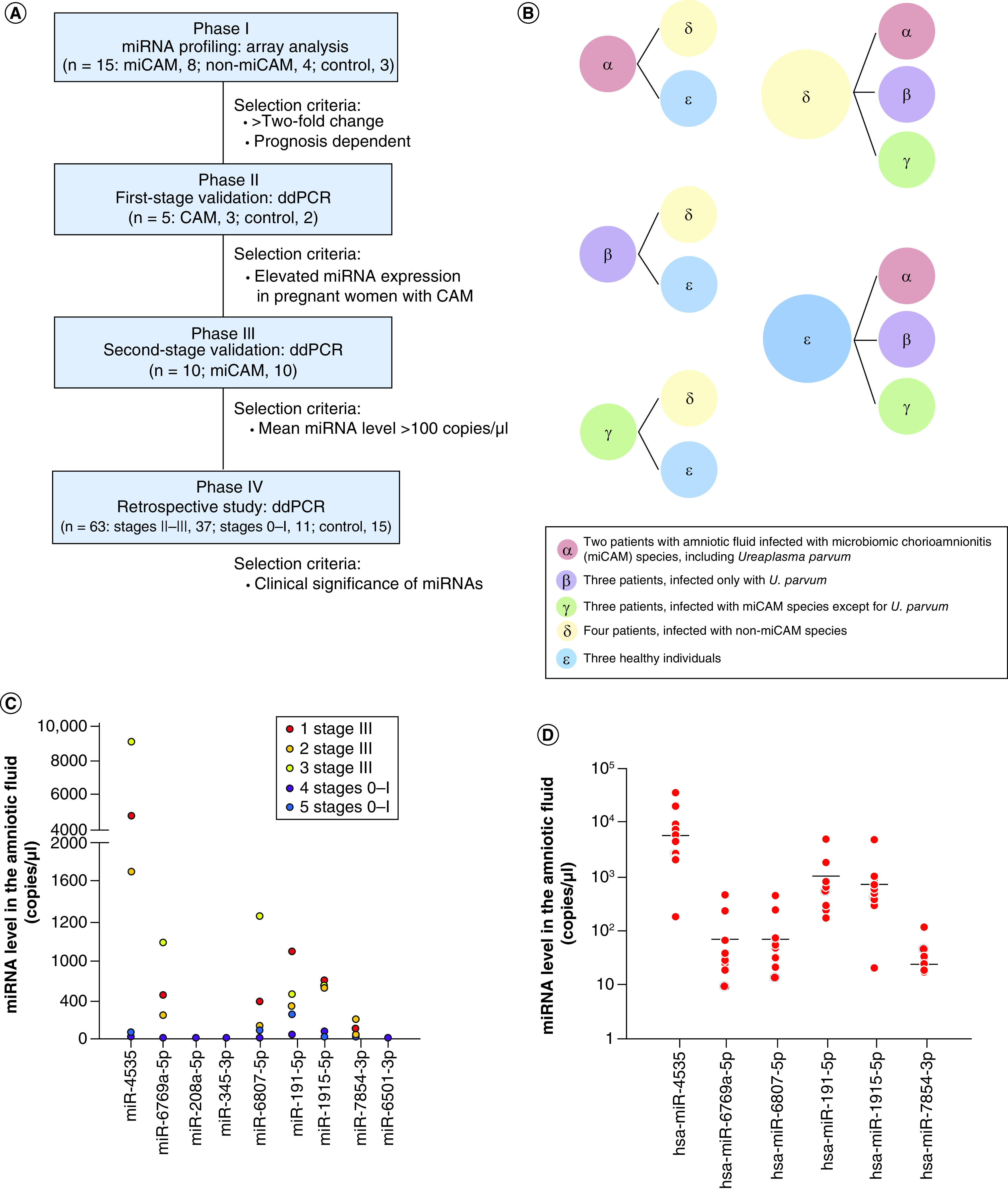
Identification of candidate miRNAs for diagnosing severe chorioamnionitis in pregnant women. **(A)** Flowchart of the study design. Predictive biomarkers in the amniotic fluid for the diagnosis of chorioamnionitis were identified using comprehensive miRNA array analysis and ddPCR™. Phases I–IV were sequentially completed. **(B)** Fifteen pregnant women were divided into five groups. The levels of nine miRNAs were identified using comprehensive miRNA array analysis of the amniotic fluid samples from pregnant women with or without chorioamnionitis. **(C)** Expression of six miRNAs in the amniotic fluid of patients with microbiomic chorioamnionitis (miCAM). Red, orange and yellow circles indicate the miRNA levels in the amniotic fluid of three pregnant women with Blanc’s classification stage III; violet and blue circles indicate the miRNA levels in the amniotic fluid of two pregnant women with Blanc’s classification stages 0 or I. **(D)** Red circles indicate the miRNA levels in 10 pregnant women with Blanc’s classification stages II–III plus miCAM. The bars indicate the median. miCAM: Microbiome chorioamnionitis.

A multiphase study was designed to identify predictive biomarkers in amniotic fluid for diagnosis of chorioamnionitis through comprehensive miRNA array analysis as well as the quantification of ddPCR, as shown in Figure 1A. After comparison of the miRNA profiles in the amniotic fluid from groups α and β or γ with those from groups δ and ε, miR-4535, miR-6769a-5p and miR-6807-5p were detected to be significantly upregulated (fold change >2.0; p < 0.05; Figure 1B & Table 1). To further select the candidate miRNAs, we examined the expression of miRNAs in the amniotic fluid among the three groups, including group α, β and γ, and group δ or ε. miR-208a-5p and miR-345-3p were found to have a more than twofold increase in expression (p < 0.05) (Figure 1B Table 1). Of the 12 patients (groups α, β, γ and δ) with Blanc’s stages II and III, two pregnant women with good prognosis and two pregnant women with poor prognosis were selected. Among the patients with a good prognosis, δ2 and δ3 had >2 days of extended hospitalization from admission to birth and 0.0 mg/dl of C-reactive protein (CRP) in the neonates after birth without the administration of antibiotics. Among the patients with poor prognosis, γ1 and γ2 had <2 days of extended hospitalization from admission to birth and 1.5 mg/dl of CRP in neonates with antibiotic treatment. Next we compared the expression of miRNAs between δ2 and δ3 and γ1 and γ2 and identified four miRNAs (miR-191-5p, miR-1915-5p, miR-7854-3p and miR-6501-3p) that showed a more than twofold increase in expression in the amniotic fluid in γ1 and γ2 (p < 0.05). Using miRNA array analyses, miR-4535, miR-6769a-5p, miR-208a-5p, miR-345-3p, miR-6807-5p, miR-191-5p, miR-1915-5p, miR-7854-3p and miR-6501-3p were identified as the candidate biomarkers for severe chorioamnionitis.

In phase II, miRNA expression was analyzed using ddPCR in the three patients with Blanc’s stage III and two patients with Blanc’s stage 0 and I. The mean white blood cell (WBC) count and CRP level in the former group were 17,533/μl and 2.93 mg/dl, respectively. The mean WBC count and CRP level in the latter group were 8,650/μl and 0.05 mg/dl, respectively. miR-4535, miR-6769a-5p, miR-6807-5p, miR-191-5p, miR-1915-5p and miR-7854-3p in the amniotic fluid exhibited a difference in three patients with Blanc’s stage III and the two patients with Blanc’s stage 0 and I (Figure 1C).

In phase III, we examined the expression of miRNAs using ddPCR in ten patients with Blanc’s stage III and identified increased expression of miR-4535, miR-191-5p and miR-1915-5p (Figure 1D). Thus we selected these three miRNAs as promising amniotic fluid biomarker candidates for severe chorioamnionitis.

Lastly, in phase IV, we analyzed the clinical significance of these three miRNAs, as explained below.

### Diagnostic significance of miRNA & 16S rDNA levels

**Figure 2.**
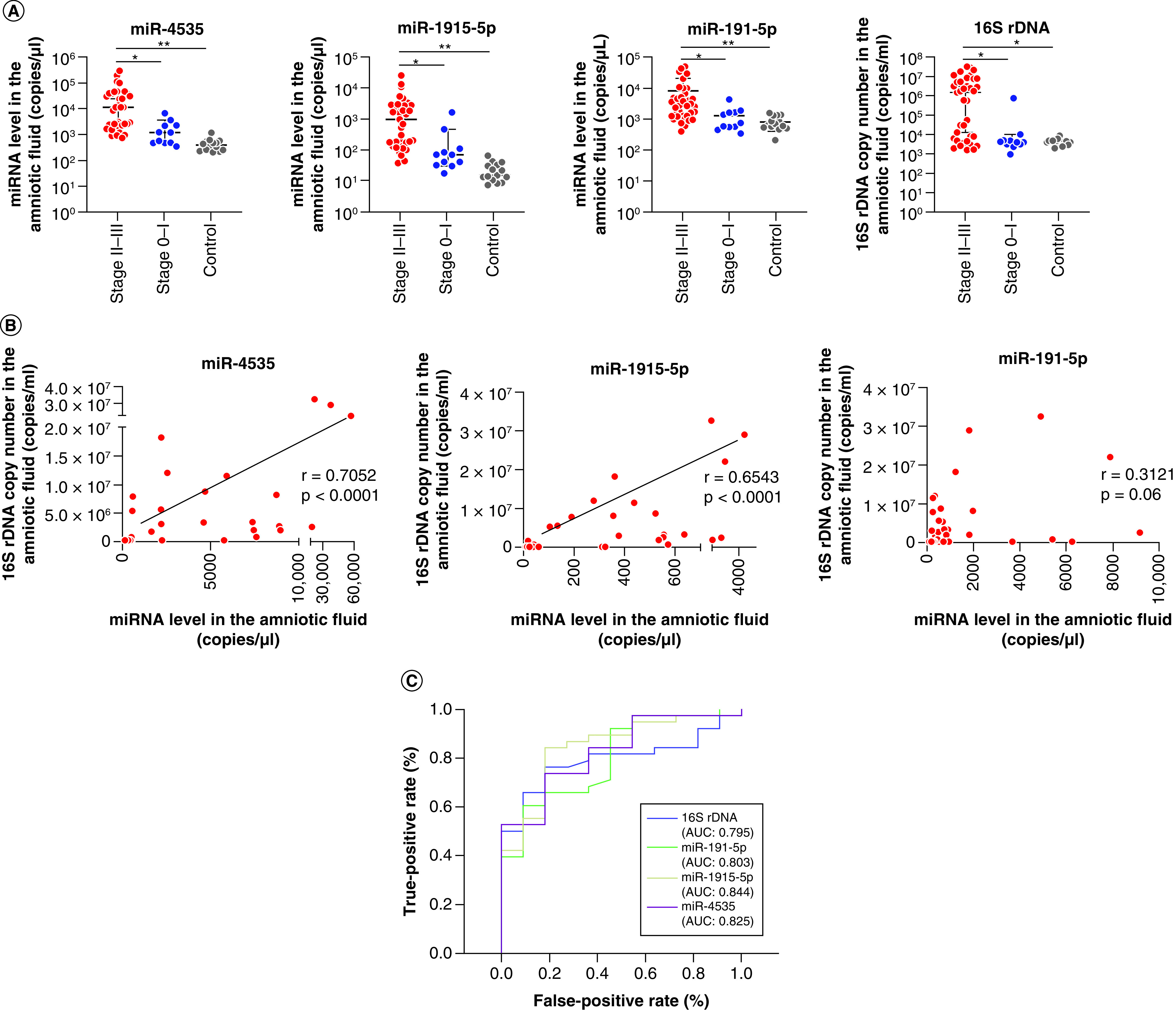
Diagnostic relationship between the level of candidate miRNAs or 16S rDNA and severe chorioamnionitis. **(A)** Expression levels of each candidate miRNA and the copy numbers of 16S rDNA in pregnant women divided into three groups: Blanc’s classification stages II–III (group II–III: n = 37, red circles); Blanc’s classification stages 0–I (group 0–I: n = 11, blue circles); and healthy pregnant women between 15 and 17 gestational weeks (control: n = 15, gray circles). Data are expressed as the median ± 95% CI. Thick and thin bars indicate the median and 95% CI, respectively. Statistical differences among the groups were assessed using Dunn’s multiple comparison test. **(B)** Correlation between miRNA expression and 16S rDNA copy number in amniotic fluid samples from 37 pregnant women with Blanc’s classification stages II–III. *r* indicates the correlation index. **(C)** Diagnostic and predictive accuracies of miRNA levels and 16S rDNA copy number for chorioamnionitis. Blue, green, yellow and purple lines indicate receiver operating characteristic curves for 16S rDNA, miR-191-5p, miR-1915-5p and miR-4535, respectively. *p < 0.01; **p < 0.0001.

To investigate the biomarkers for the predictive diagnosis of severe chorioamnionitis, we examined the three miRNAs and the quantified 16S rDNA. The expression levels of miR-4535, miR-191-5p and miR-1915-5p in the amniotic fluid were significantly augmented in groups II–III, compared with those in group I and the control group (all p < 0.01). In groups II–III, the copy number of 16S rDNA in the amniotic fluid was also significantly augmented compared with group I and the control group (p < 0.01; Figure 2A); the expression of miR-4535 and miR-1915-5p was significantly correlated with the copy number of 16S rDNA in the amniotic fluid (p < 0.01) whereas that of miR-191-5p was not (Figure 2B).

Next we calculated the AUC, Youden index, cutoff value, sensitivity and specificity for the ROC curve to predict severe chorioamnionitis from the amniotic fluid. The AUC values for miR-4535, miR-1915-5p and miR-191-5p indicated higher diagnostic accuracy than those for 16S rDNA (Figure 2C). Regarding miR-1915-5p expression, the Youden index and cutoff value were 0.660 and 115.864 copies/μl, respectively. Additionally, the sensitivity and specificity were 84.2 and 81.8%, respectively. Regarding miRNA expression, the Youden index, cutoff value, sensitivity and specificity were 0.555, 2320.962 copies/μl, 73.7% and 81.8%, respectively, for miR-4535; and 0.514, 1982.604 copies/μl, 60.5% and 90.9%, respectively, for miR-191-5p. Regarding the copy number of 16S rDNA, the Youden index, cutoff value, sensitivity and specificity were 0.581, 5637.5 copies/ml, 73.7% and 81.8%, respectively. These data indicate that miRNA expression is a more accurate diagnostic biomarker for severe chorioamnionitis than the quantification of infection intensities. In addition, miR-4353 and miR-1915-5p may be linked to the extent of infection.

### Association of miRNAs & 16S rDNA in patients with miCAM

**Figure 3.**
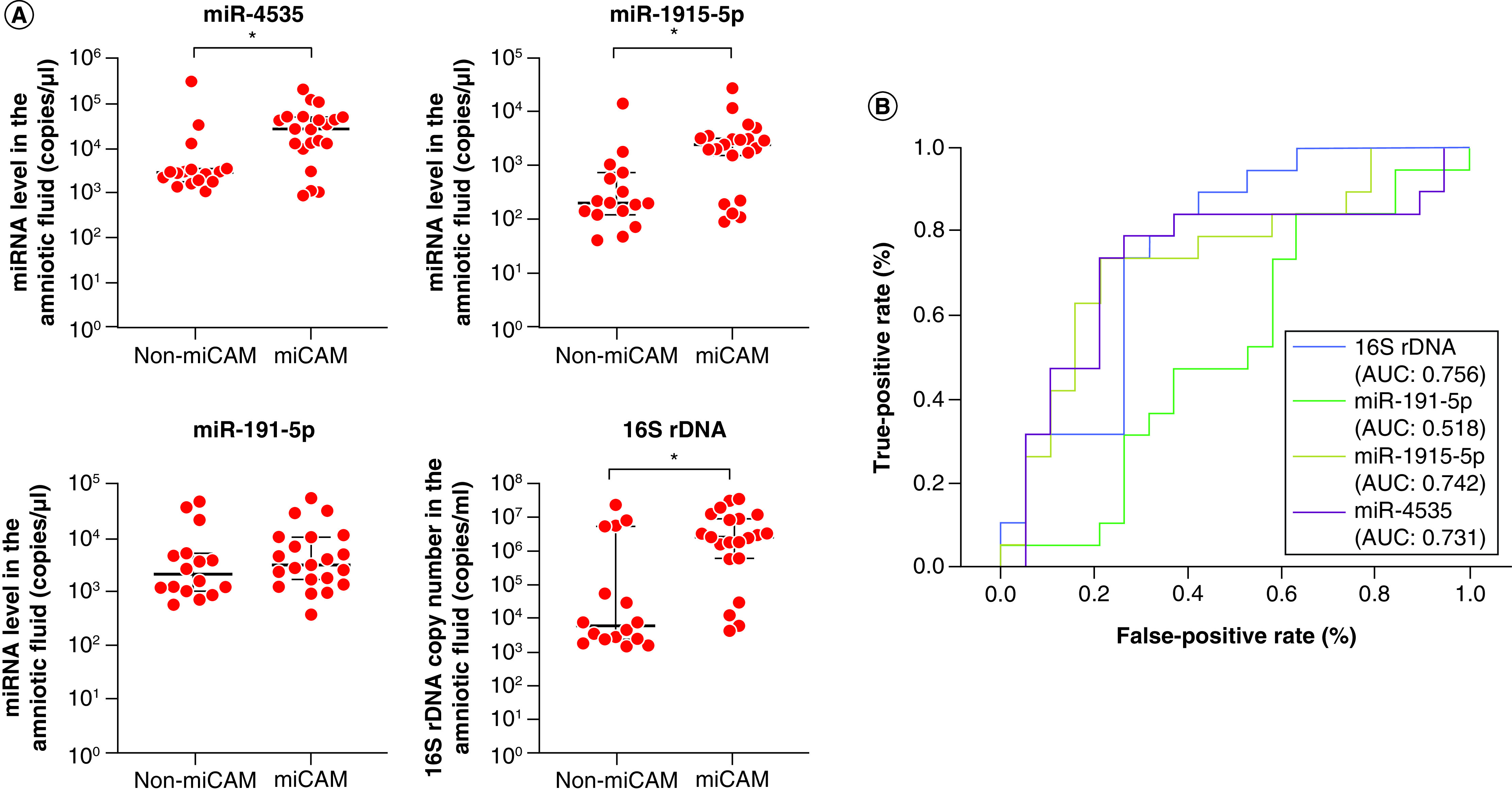
Expression of candidate miRNAs and quantification of 16S rDNA in patients with microbiomic chorioamnionitis. **(A)** Comparison of miR-4535, miR-1915-5p and miR-191-5p levels and 16S rDNA copy numbers in the amniotic fluid from pregnant women with microbiomic chorioamnionitis (miCAM) (n = 21) and without miCAM (non-miCAM) (n = 16). Data are expressed as the median ± 95% CI. Thick and thin bars indicate the median and 95% CI, respectively. **(B)** Diagnostic and predictive accuracies of miRNA expression and 16S rDNA copy number for chorioamnionitis and miCAM. Blue, green, yellow and purple lines indicate receiver operating characteristic curves for 16S rDNA, miR-191-5p, miR-1915-5p and miR-4535, respectively. *p < 0.05. CRP: C-reactive protein; miCAM: Microbiome chorioamnionitis; WBC: White blood cell.

To gain insight into the association of the biomarkers with miCAM, we compared the expression of three miRNAs and the quantification of 16S rDNA in groups II–III. In groups II–III, 21 of 37 patients had miCAM, and the remaining 16 had infection without miCAM (non-miCAM). The expression of miR-4535 and miR-1915-5p and the copy number of 16S rDNA in the miCAM group was significantly increased compared with that in the non-miCAM group (p < 0.01) (Figure 3A). Regarding miR-191-5p expression, no significant difference was found in pregnant women with miCAM compared with those without miCAM (Figure 3A). The AUC, Youden index, cutoff value and detection of sensitivity and specificity for the ROC curve were calculated for the three miRNAs and 16S rDNA. The AUC values for miR-4535 and miR-1915-5p and the copy number of 16S rDNA showed higher diagnostic accuracy compared with those for miR-191-5p (Figure 3B). Regarding the expression of miRNAs, the Youden index, cutoff value, sensitivity and specificity were 0.526, 5858.371 copies/μl, 78.9% and 73.7 %, respectively, for miR-4535; and 0.526, 1232.255 copies/μl, 73.7% and 78.9%, respectively, for miR-1915-5p. For 16S rDNA copy number, the Youden index, cutoff value, sensitivity and specificity were 0.474, 1,074,700 copies/ml, 73.7% and 73.7%, respectively. Regarding the expression of miR-191-5p, the Youden index, cutoff value, sensitivity and specificity were 0.211, 1185.973 copies/μl, 84.2% and 36.6%, respectively. Overall, these results suggested that the index for infection and miRNA expression involved in miCAM might serve as valuable biomarkers for severe chorioamnionitis.

### Clinical significance of miRNAs & 16S rDNA in the amniotic fluid of patients with severe chorioamnionitis

**Figure 4.**
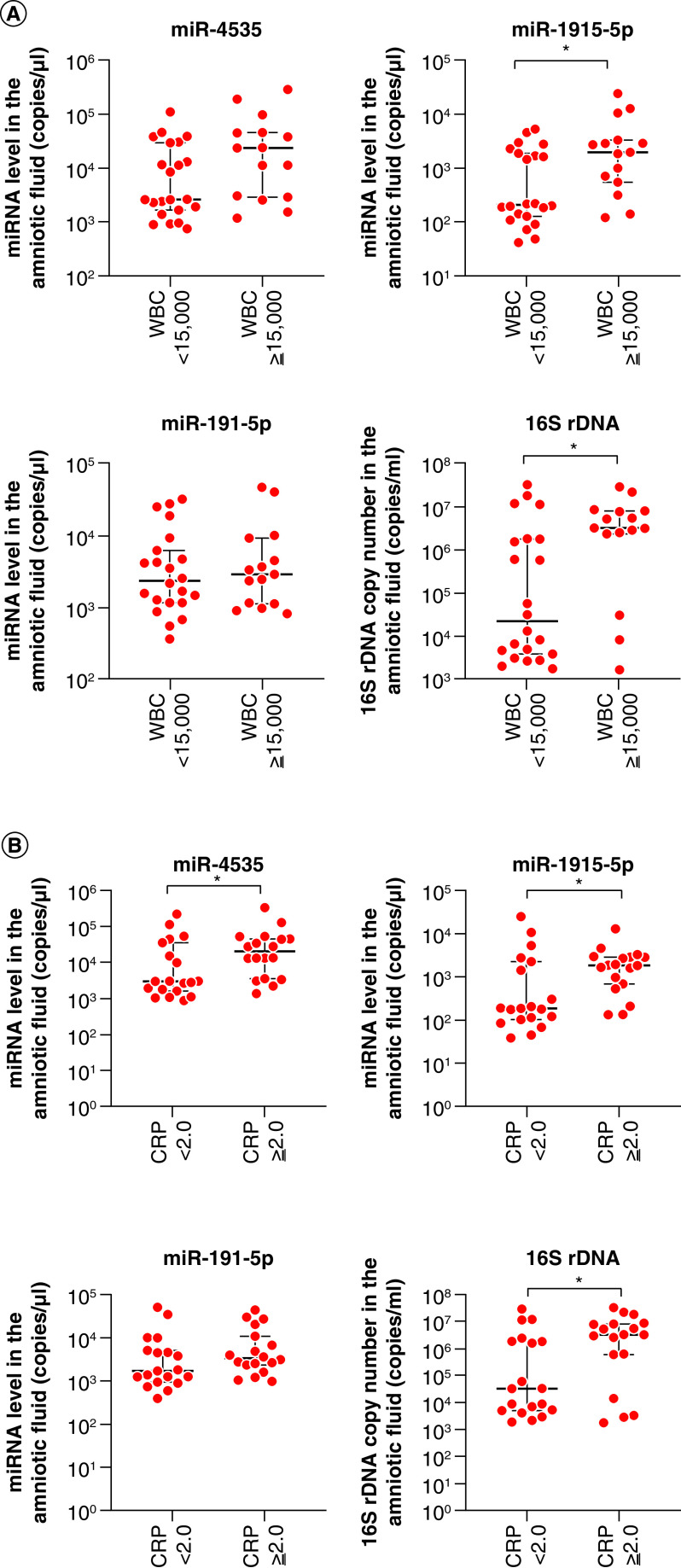
Relationship between (A) maternal white blood cell counts or (B) serum CRP and miRNA levels or 16S rDNA copy number in the amniotic fluid of 37 pregnant women with Blanc’s classification stages II–III. Data are expressed as the median ± 95% CI. Thick and thin bars indicate the median and 95% CI, respectively. *p < 0.05. CRP: C-reactive protein; WBC: White blood cell.

To elucidate the significance of the putative biomarkers in maternal and fetal inflammation, we examined the expression of the three miRNAs and the copy number of 16S rDNA in groups II–III, in which 15 had high WBC counts (>15,000/μl), whereas the other 22 had low WBC counts (<15,000/μl). The expression of miR-1915-5p and the copy number of 16S rDNA in the amniotic fluid were significantly increased in patients with high WBC count (p < 0.05; Figure 4A). No significant differences in miR-4535 or miR-191-5p were observed (Figure 4A).

In addition, groups II–III were divided into two based on CRP levels in the maternal serum: 18 had a CRP level of >2.0 mg/ml, and 19 had CRP level of <2.0 mg/ml. The expression of miR-4535 and miR-1915-5p and the copy number of 16S rDNA in the amniotic fluid significantly increased in patients with high CRP levels (p < 0.05; Figure 4B). Regarding the expression of miR-191-5p, no significant difference was observed between the two groups (Figure 4B). These results indicate that the expression of miR-4535 or miR-1915-5p and the quantification of 16S rDNA might be related to maternal inflammation.

**Figure 5.**
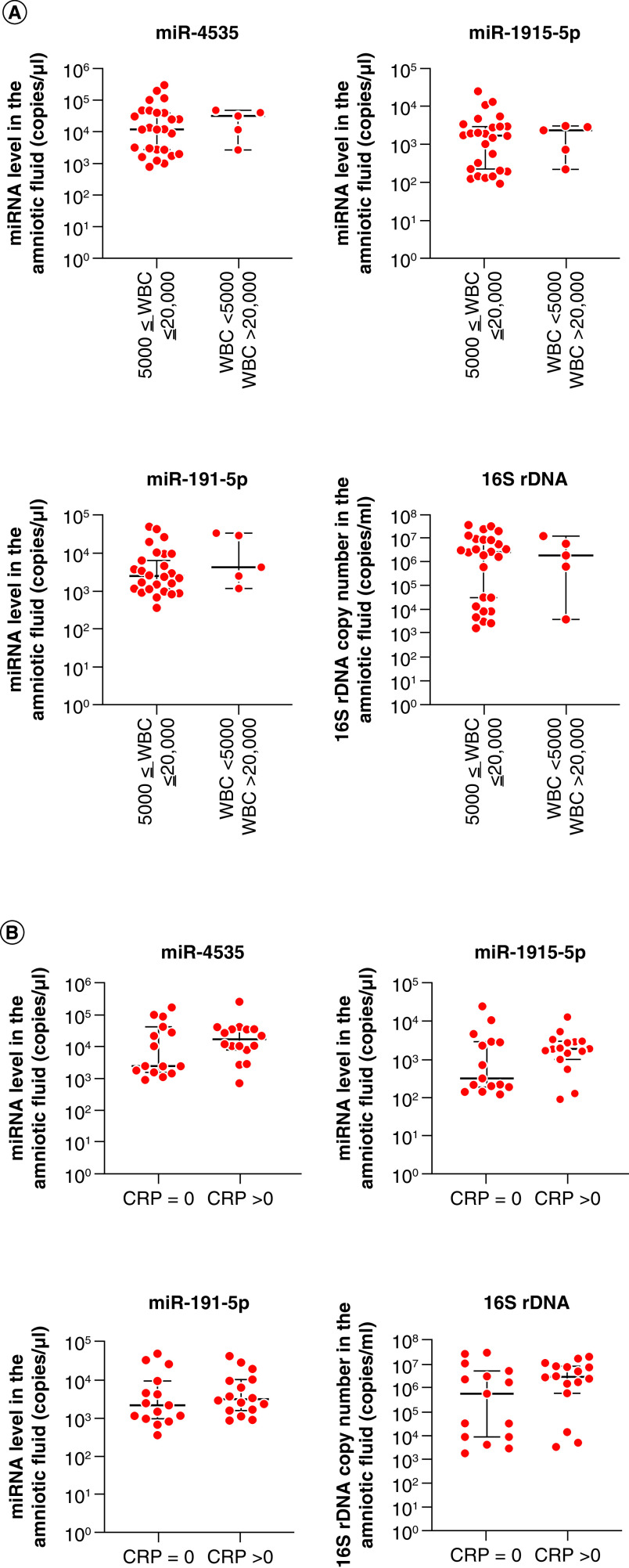
Relationship between (A) fetal white blood cell counts or (B) serum CRP and miRNA levels or 16S rDNA copy number in the amniotic fluid of pregnant women with Blanc’s classification stages II–III. Data are expressed as the median ± 95% CI. Thick and thin bars indicate the median and 95% CI, respectively. *p < 0.05. CRP: C-reactive protein; WBC: White blood cell.

To estimate the clinical role of putative biomarkers in fetal inflammation, 31 neonates in groups II–III were analyzed and divided into two groups: 26 had a WBC count of 5000–20,000/μl, and 5 had a WBC count of <5000 or >20,000/μl. No significant differences in the expression of miR-4535, miR-1915-5p or miR-191-5p, or the copy number of 16S rDNA, in the amniotic fluid were detected between the two groups (Figure 5A). In addition, groups II–III were divided into two groups based on the serum CRP level in the neonates: 15 had a CRP level of 0.0 mg/dl and 16 had a CRP level of >0.0 mg/dl. There were no significant differences in the expression of miR-4535, miR-1915-5p or miR-191-5p, or the copy number of 16S rDNA, between these two groups (Figure 5B).

To further evaluate the relationship between the putative biomarkers and fetal inflammation, we examined the incidence of serious inflammation in the highest quartile of the expression of the three miRNAs and the copy number of 16S rDNA. Patients with a CRP level of 0.0 mg/ml and a WBC count of 5000–20,000/μl were classified as having no definite inflammation. In contrast, when the CRP level was >0.0 mg/ml and the WBC count was <5000 or >20,000/μl, patients were suspected to have serious inflammation mediated by sepsis. The third quartile (>75%) of the miR-4535 and miR-1915-5p expression was significantly associated with suspicious septic cases in the neonates (Table 2). miR-191-5p and 16s rDNA were not recognized as important biomarkers for neonatal infection. These results indicate that extremely high expression of miR-4535 and miR-1915-5p in the amniotic fluid can be considered as a novel biomarker for neonatal infection.

**Table 2.** Association of neonatal inflammatory indicators between newborns with the third quartile of miRNA levels in the amniotic fluid.

	High inflammatory indicators^†^	Low inflammatory indicators^‡^	p-value^§^
miR-4535 (copies/μl) ≥2665.4 <2665.4	15 1	8 7	0.0102
miR-1915-5p (copies/μl) ≥212.94 <212.94	17 2	6 6	0.0144
miR-191-5p (copies/μl) ≥1181.4 <1181.4	15 3	8 5	0.1712
16S rDNA (copies/ml) ≥21,945 <21,945	15 3	8 5	0.1712

n = 31 for pregnant women with Blanc’s classification stages II–III.

† Neonatal CRP level > 0 mg/dl after birth, or white blood cell count <5000 or >25,000/μl after birth.

‡ Neonatal CRP level = 0 mg/dl after birth and white blood cell count 5000–25,000/μl after birth.

§ Statistical analysis was performed using the Pearson’s χ-square test.

### Assessment of miRNA levels in the serum as biomarkers for severe chorioamnionitis

**Figure 6.**
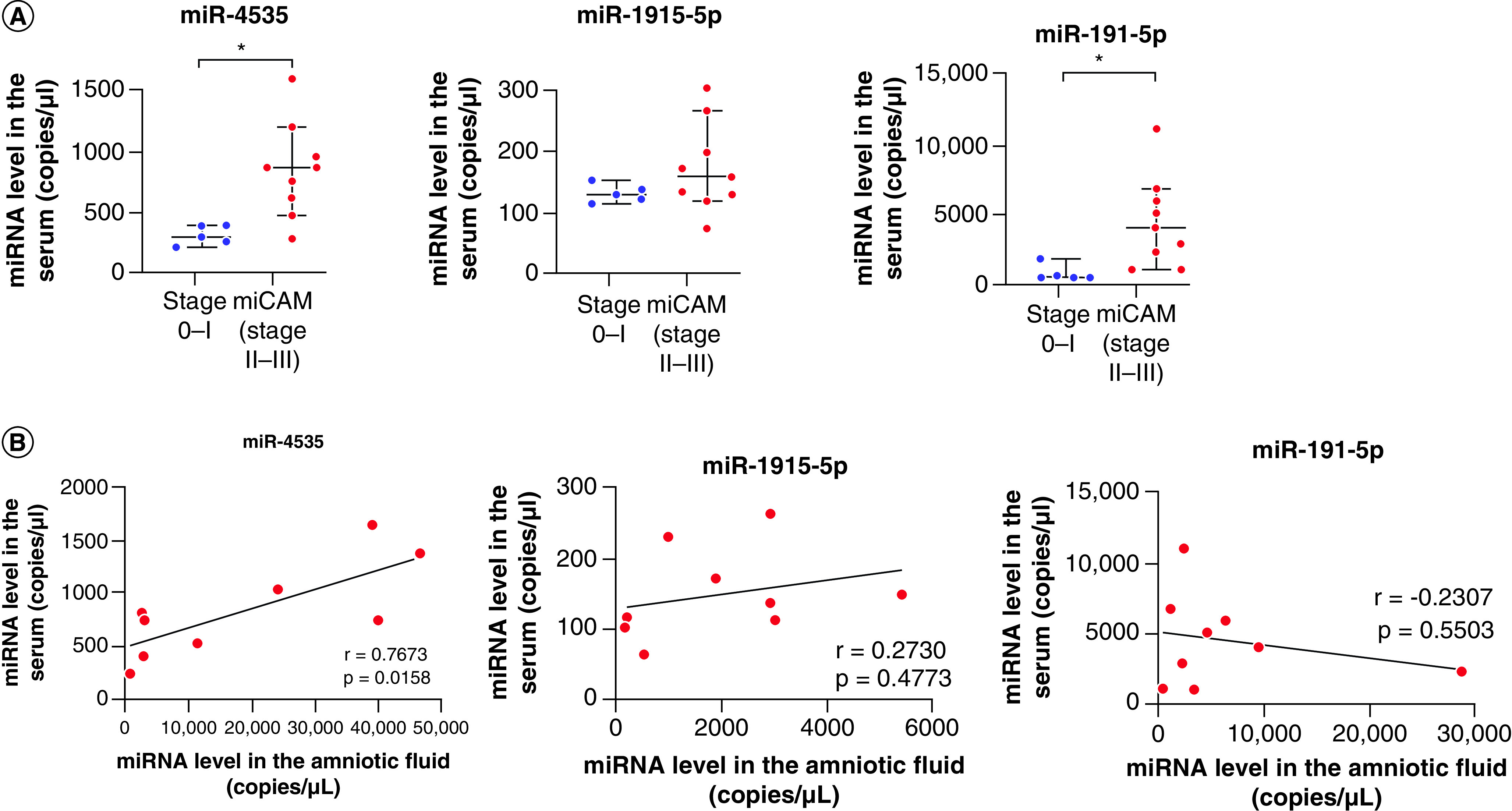
Clinical significance of candidate miRNAs in the serum of patients with severe chorioamnionitis. **(A)** Levels of miR-4535, miR-1915-5p and miR-191-5p in the serum of pregnant women with Blanc’s classification stages II–III and microbiomic chorioamnionitis (n = 9) and those with Blanc’s classification stages 0–I (n = 5). Data are expressed as the median ± 95% CI. Thick and thin bars indicate the median and 95% CI, respectively. **(B)** Correlation of the expression of miR-4535, miR-1915-5p and miR-191-5p in the amniotic fluid (x-axis) and serum (y-axis) of pregnant women with Blanc’s classification stages II–III and miCAM (n = 9). *r* indicates the correlation index. *p < 0.05. miCAM: Microbiome chorioamnionitis.

To investigate the potential serum biomarker role of the three miRNAs, we examined the presence of miR-4535, miR-1915-5p, miR-191-5p and 16S rDNA in the serum using ddPCR. In nine patients exhibiting Blanc’s classification stages II–III with miCAM, the expression of miR-4535 and miR-191-5p in the serum was significantly increased compared with five patients with Blanc's classification stages 0–I (p < 0.05; Figure 6A). The 16S rDNA was not detected in the serum of patients with Blanc’s classification stages II–III plus miCAM. In nine patients with Blanc’s classification stages II–III plus miCAM, the expression of miR-4535 in the serum was significantly correlated with that in amniotic fluid (p < 0.05), whereas no significant relationship was observed between the serum and amniotic fluid for miR-1915-5p or miR-191-5p expression (Figure 6B). These results support our hypothesis that the expression of miR-4535 in the serum might serve as a predictive factor for the diagnosis of severe chorioamnionitis.

### Target genes of candidate miRNAs

We investigated the target genes by drawing Venn diagrams of the target of each candidate miRNA. There were six unique intersecting miRNA–mRNA pairs for hsa-miR-4535: *CD44*, *LHFPL3*, *KDM1B*, *RNF19A*, *C19orf82* and *FKBP4*. There were seven unique intersecting miRNA–mRNA pairs for hsa-miR-1915-5p: *TFE3*, *RBM39*, *SYT4*, *B3GALT5*, *ZNF83*, *TMEM115* and *DIO2*. There were four unique intersecting miRNA–mRNA pairs for hsa-miR-191-5p: *TMOD2*, *SATB1*, *ZBTB34* and *PLCD1*.

## Discussion

In this study we demonstrated that miR-4535, miR-1915-5p and miR-191-5p were abundantly present in the amniotic fluid of patients with severe chorioamnionitis and that the expression level of these miRNAs provided a more accurate diagnosis of severe chorioamnionitis than that of 16S rDNA. In addition, the expression of miR-4535 and miR-1915-5p were associated with maternal and fetal inflammatory outcomes. The expression levels of miR-4535 in the amniotic fluid and serum were significantly correlated, suggesting that the measurement of miR-4535 levels in the serum might be useful for the diagnosis of severe chorioamnionitis. Thus we have shown that miR-4535 and miR-1915-5p levels in the amniotic fluid are promising biomarkers for the diagnosis of severe chorioamnionitis.

Intrauterine infection and inflammation are well-documented causes of preterm labor with intact fetal membranes [48,49]. Additionally, adverse perinatal outcomes are associated with infection and inflammation [10,50]. Despite a negative amniotic fluid culture, pregnant women in preterm labor often exhibit intra-amniotic inflammation [51–53]. Using PCR amplification, the prokaryotic 16S rRNA or its corresponding DNA (rDNA) in the amniotic fluid has been found in pregnant women, even those in preterm labor with negative amniotic fluid cultures [12,18,54]. ddPCR has many advantages over real-time PCR, including the capability to obtain accurate quantification without external references and high robustness that allows variations in efficiency [55,56]. In this study no subjects among our patient cohort showed negative results in ddPCR analysis. Additionally, all subjects showed >1000 copies/ml of 16S rDNA. Therefore we showed that the assessment of PCR products using ddPCR was feasible for diagnosing chorioamnionitis. However, in terms of specificity and sensitivity, detecting the miR-4535 level was better than detecting 16S rDNA, suggesting that inflammation involves the response of extra-amniotic compartments (e.g., the decidua, membranes and placenta) against microbial invasion [57–59]. In the present study, miR-548, miR-223 and miR-338, which are significantly associated with chorioamnionitis, were little expressed according to the miRNA array analysis and were not picked up in the screening. Reportedly, the amount of each of these miRNAs in women with chorioamnionitis is a few times that in pregnant women without chorioamnionitis [46,47]. In women with chorioamnionitis, the amounts of miR-4535, miR-1915-5p and miR-191-5p in amniotic fluid were found to be more than ten-times that in pregnant women without chorioamnionitis. In principle, the majority of miRNAs in amniotic fluid are generated from fetal and maternal tissues, such as amniotic membrane, chorionic membrane, placental tissues, fetal inflammatory cells and maternal inflammatory cells; therefore it is plausible that miR-4535, miR1915-5p or miR-191-5p in amniotic fluid may be excreted from many types of cells, including the cells of amniotic membrane. Therefore quantifying 16S rDNA, miR-4535 and miR-1915-5p levels using ddPCR and detecting miCAM using PCR or next-generation sequencing are promising steps to accurately and rapidly determine Blanc’s classification and chorioamnionitis in pregnant women before delivery.

The intensity and frequency of fetal inflammatory responses continuously increase according to the progression of inflammation in the umbilical cord [60,61]. The expression of miR-4535 and miR-1915-5p in the amniotic fluid was found to be related to the fetal and maternal inflammatory responses. Intense inflammatory responses are mediated by various cytokines or inflammatory products, including IL-1β, TNF-α, IL-6, PAF, IFN-γ, PGE2, MMP-1 and MMP-2 [19]. In particular, the fetal inflammatory response syndrome is characterized by systemic inflammation and elevated fetal plasma IL-6 levels [62]. Excess IL-6 levels may lead to neonatal sepsis, periventricular leukomalacia and necrotizing enterocolitis [63]. In general, IL-6 is a proinflammatory cytokine with functional pleiotropy which plays an important role in host defence; however, uncontrolled or persistent IL-6 production is involved in the development of various inflammatory disorders [64,65]. Furthermore, its regulation is associated with many miRNAs that may also be involved in excess IL-6 secretion [66,67]. For instance, miR-let7 and miR-146a negatively regulate IL-6 expression via inhibition of the signal transducer for IL-6 expression and the activator of transcription-3 or NF-κB function [68,69]. Thus further studies investigating the relationship between miR-4535 or miR-1915-5p levels and inflammatory responses might yield novel key molecules in fetal inflammatory systemic syndrome.

In animal study experiments, lipoprotein multiple-banded antigen (MBA), which is isolated from *U. parvum*, has powerful virulence properties for the induction of preterm birth [70]. The expression of MBA size variants is linked to activating inflammatory responses including enhanced NF-κB and increased expression of TNF-α, and the size variant of MBA derived from *U. parvu*m modulates the host immune response [71-73]. In this study, miR-4535 and miR-1915-5p in amniotic fluid were highly expressed in patients with miCAM compared with those in patients without infection. Accordingly, it is plausible that the infection of miCAM including *U. parvum* may result in a further elevated amount of miR-4535 and miR-1915-5p in severe chorioamnionitis. The biological role of elevated levels of miR-4535 or miR-1915-5p is unclear. In contrast, miR-1915-5p is involved in many biological processes, including epithelial–mesenchymal transition, inhibition of phosphatidylinositol 3-kinase activity, repair of adult renal progenitor cells and the apoptotic response to DNA damage via p53 induction [74,75]. Additionally, miR-4535, an intronic miRNA with no other family member, is found only in humans and its function has been rarely reported (miRbase version 21). To the best of our knowledge, neither miR-4535 nor miR-1915-5p has been associated with infection or inflammation; this is the first report to associate miR-4535 and miR-1915-5p with chorioamnionitis. This suggests that bacterial infection in the amniotic fluid might be a different event generated in an unusual environment, such as a completely closed cavity with infection, compared with pulmonary infection, gut infection and infections in other organs.

Through *in silico* analysis, 17 genes that were putatively associated with the three miRNAs (miR-4535, miR-1915-5p and miR-191-5p) were selected. Of these, *CD44*, *KDM1B*, *FKBP4*, *TEF*, *SYT4*, *SATB1* and *PLCD1* are reported to be involved in the inflammatory reaction [76–82]; for the remaining ten genes, there have been no reports concerning inflammation or infection. In principle, CD44 and SYT4 are membrane proteins, and the other molecules are nuclear proteins or transcriptional factors. SYT4 is mainly expressed in neurons, while CD44 is displayed on mesenchymal stem cells, which contribute to the repair of the amnion epithelial cells. Furthermore, CD44 binds to hyaluronic acid, leading to the modulation of proliferation, migration and angiogenesis. As CD44 is required in the main phase of wound healing [83], the enhanced expression of miR-4535 might probably be compensated for fixing the damaged amnion in the inflammatory response.

To date, several studies have identified maternal blood biomarkers that are predictive of histological chorioamnionitis, including various host proteins secreted in response to microbial pathogens, such as serum CRP, MMP-9, IL-6, S100 calcium-binding proteins A8/A9 and IGFBP-1 [84,85]. These molecules have also been observed in the amniotic fluid in women with severe chorioamnionitis [19–21]. In this study we showed that the miR-4535 level in serum was significantly correlated with that in amniotic fluid in women with severe chorioamnionitis, suggesting that the alteration in amniotic fluid can be monitored via the expression of miR-4535 in the serum. Thus miR-4535 may be the first nucleic acid to be identified as a predictive biomarker in the serum for chorioamnionitis [86]. Further studies are required to elucidate the biological role of the predictive biomarkers in the serum that we have identified in this study.

## Conclusion

Older pregnant women are at higher risk for premature birth and chorioamnionitis. To protect newborns against the fetal inflammatory response syndrome, the early diagnosis of chorioamnionitis is essential. Here we have demonstrated that the levels of 16S rDNA, miR-4353 and miR-1915-5p in the amniotic fluid may be potential biomarkers for the diagnosis of chorioamnionitis.

## Future perspective

Accumulating clinical evidence for miR-4535 and miR-1915-5p can provide huge information on the biological roles of these two miRNAs in chorioamnionitis hereafter. In the near future it is promising that nucleic acid medicine for miR-4535 or miR-1915-5p would ameliorate the cytokine storm in perinatal diseases including chorioamnionitis and fetal inflammatory response syndrome.

**Summary points**

- Increasing average age of women at childbirth is associated with an increased incidence of premature birth with severe chorioamnionitis.
- Chorioamnionitis is associated with a range of morbidities including premature birth, neonatal sepsis, brain damage and necrotizing enterocolitis before delivery.
- To date, only a few accurate biomarkers in the amniotic fluid have been identified for diagnosing chorioamnionitis.
- miRNAs have great potential as diagnostic and prognostic biomarkers of various diseases.
- This study is the first to report useful biomarkers for the prediction of severe chorioamnionitis.
- The expression of miR-4535 and miR-1915-5p was significantly elevated in patients with severe chorioamnionitis.
- Receiver operating characteristic curve analysis indicated that the levels of miR-4535 and miR-1915-5p provide a more accurate diagnosis for severe chorioamnionitis than the levels of 16S rDNA.
- The expression levels of miR-4535 and miR-1915-5p in the amniotic fluid were associated with maternal and fetal inflammatory responses.
- The expression levels of miR-4535 and miR-191-5p in the serum were significantly increased in patients with severe chorioamnionitis.
- A significant correlation was found between the expression of miR-4535 in the amniotic fluid and serum, whereas that of miR-1915-5p or miR-191-5p in the serum was not correlated with the expression in amniotic fluid.
